# Radiation education for nurses working at middle-sized hospitals in Japan

**DOI:** 10.1093/jrr/rrz049

**Published:** 2019-07-12

**Authors:** Masato Nagatomi, Takumi Yamaguchi, Tetsuko Shinkawa, Yasuyuki Taira, Hideko Urata, Makiko Orita, Noboru Takamura

**Affiliations:** 1 Division of Disaster and Radiation Medical Sciences, Nagasaki University Graduate School of Biomedical Sciences, 1-12-4 Sakamoto, Nagasaki, Japan; 2 Department of Global Health, Medicine and Welfare, Atomic Bomb Disease Institute, Nagasaki University, 1-12-4 Sakamoto, Nagasaki, Japan

Eight years have passed since the accident at Fukushima Daiichi Nuclear Power Station. After the accident, the insufficient knowledge on radiation in medical personnel, including nurses was revealed [1], which caused some embarrassment [2]. Previously, we proposed that nurses should have roles of risk communication regarding radiation health effects and radiation protection during nuclear emergencies [3]. On that basis, nurses should gain fundamental knowledge about radiation [4].

However, in Japan, most nursing students have not been educated on radiation, and several studies have reported insufficient knowledge about radiation in nurses [5, 6]. Although most hospitals in Japan have established a training system for nurses, it usually focuses on basic skills related to patient care, especially in middle-sized hospitals, and training about radiation is usually insufficient. In this study, we investigated the intention of nurses to work at the radiological department of a middle-scale hospital and on the level of training on radiation that is provided.

We sent questionnaires to 801 nurses working at middle-sized hospitals (200−500 beds) in Nagasaki Prefecture, Japan. In total, 661 of 801 nurses (82.5%) answered the questionnaire. After excluding 114 nurses who failed to respond fully, 547 were included in the analysis. In the questionnaires, we included questions about nurses’ intentions of work at the radiological department and of participate in a training course about radiation. We asked nurses to respond to each question with ‘Yes’ or ‘No’. In addition, we also asked them to explain in an open-ended manner why they had answered ‘Yes’ or ‘No’ to each question. We analyzed qualitative data using the ‘KH coder’ developed by Ko-ichi Higuchi [7], which is a free text-mining software used to analyze text-type materials. We extracted specific words and the number of times they occurred in the questionnaire. This study was approved by the ethical committee of Nagasaki University Graduate School of Biomedical Sciences (No.17120426).

Of 547nurses, 470 (85.9%) answered that they did not want to work at the radiological department. Extracted words and the reasons regarding their intention to work, or not, at a radiological department are shown in Table 1. They frequently answered ‘I don’t want to be exposed to radiation’, or ‘Because of lack of knowledge on radiation’, which suggested that nurses do not want to engage at a radiological department because they have little knowledge about radiation. Furthermore, it was revealed that some nurses intended not to work at a radiological department due to anxiety about the effect of radiation on the health of infants.

**Table 1.** Extracted words and examples of responses to the question on intention to work at a radiological department

Extracted word	Frequency of word appearance	Example of a sentence
Exposure	38	I don’t want to be exposed to radiation
Knowledge	28	Because of lack of knowledge about radiation
Interest	27	I have no interest
Work	27	I want to work in a general ward
Radiation	24	Radiation does not have a good image
Think	22	I think that I experience quite a few effects of radiation
Pregnancy	18	Because I want to become pregnant
Hope	15	I hope not to work with radiation if possible
Especially	15	I have never thought especially about it
Hospital ward	15	I want to work in a hospital ward

On the other hand, 540 of 547 nurses (94.7%) recognized the necessity of education about radiation. Nurses who expressed their intention to participate in the training on radiation expressed their ‘lack of knowledge on radiation’ with responses such as ‘I don’t have enough knowledge’ and ‘I need minimum knowledge about radiation’. Additionally, some nurses responded that ‘I want to learn about radiation and radiation technology that can be used at daily work’.

Many nurses working at middle-sized hospitals recognized their insufficient knowledge about radiation. Anxieties about radiation due to insufficient knowledge by nurses may influence their intention to work, or not, at a radiological department. It is expected that the anxieties of nurses can be reduced by education that provides the correct knowledge on radiation.

## CONFLICT OF INTEREST

None of the authors have a conflict of interest.
